# Switchgrass (*Panicum virgatum *L.) polyubiquitin gene (*PvUbi1 *and *PvUbi2*) promoters for use in plant transformation

**DOI:** 10.1186/1472-6750-11-74

**Published:** 2011-07-11

**Authors:** David GJ Mann, Zachary R King, Wusheng Liu, Blake L Joyce, Ryan J Percifield, Jennifer S Hawkins, Peter R LaFayette, Barbara J Artelt, Jason N Burris, Mitra Mazarei, Jeffrey L Bennetzen, Wayne A Parrott, Charles N Stewart

**Affiliations:** 1 Department of Plant Sciences, University of Tennessee, Knoxville, TN 37996, USA; 2 Department of Crop and Soil Sciences, University of Georgia, Athens, GA 30602, USA; 3 Department of Genetics, University of Georgia, Athens, GA 30602, USA; 4 The BioEnergy Science Center, Oak Ridge National Laboratory, Oak Ridge, TN 37831-6026, USA

## Abstract

**Background:**

The ubiquitin protein is present in all eukaryotic cells and promoters from ubiquitin genes are good candidates to regulate the constitutive expression of transgenes in plants. Therefore, two switchgrass (*Panicum virgatum *L.) ubiquitin genes (*PvUbi1 *and *PvUbi2*) were cloned and characterized. Reporter constructs were produced containing the isolated 5' upstream regulatory regions of the coding sequences (i.e. *PvUbi1 *and *PvUbi2 *promoters) fused to the *uidA *coding region (*GUS*) and tested for transient and stable expression in a variety of plant species and tissues.

**Results:**

*PvUbi1 *consists of 607 bp containing *cis*-acting regulatory elements, a 5' untranslated region (UTR) containing a 93 bp non-coding exon and a 1291 bp intron, and a 918 bp open reading frame (ORF) that encodes four tandem, head -to-tail ubiquitin monomer repeats followed by a 191 bp 3' UTR. *PvUbi2 *consists of 692 bp containing *cis*-acting regulatory elements, a 5' UTR containing a 97 bp non-coding exon and a 1072 bp intron, a 1146 bp ORF that encodes five tandem ubiquitin monomer repeats and a 183 bp 3' UTR. *PvUbi1 *and *PvUbi2 *were expressed in all examined switchgrass tissues as measured by qRT-PCR. Using biolistic bombardment, *PvUbi1 *and *PvUbi2 *promoters showed strong expression in switchgrass and rice callus, equaling or surpassing the expression levels of the CaMV *35S, 2x35S, ZmUbi1*, and *OsAct1 *promoters. GUS staining following stable transformation in rice demonstrated that the *PvUbi1 *and *PvUbi2 *promoters drove expression in all examined tissues. When stably transformed into tobacco (*Nicotiana tabacum*), the *PvUbi2+3 *and *PvUbi2+9 *promoter fusion variants showed expression in vascular and reproductive tissues.

**Conclusions:**

The *PvUbi1 *and *PvUbi2 *promoters drive expression in switchgrass, rice and tobacco and are strong constitutive promoter candidates that will be useful in genetic transformation of monocots and dicots.

## Background

Genetic transformation is an important tool for crop improvement and research in genetics. The transformation of bioenergy crops with genes that alter plant development rate, growth habit, cell wall structure and/or composition has been deemed a promising approach to reduce cell wall recalcitrance (i.e., resistance to enzymatic degradation during saccharification) or to increase biomass yields [1-4]. Switchgrass (*Panicum virgatum *L.), a C4 perennial grass species native to the prairies of North America, is a candidate lignocellulosic feedstock for bioenergy [5-7]. The development of tissue culture and transformation systems has led to significant breakthroughs and applications in switchgrass biotechnology [8-11]. Recent increases in transformation efficiency [12], along with recent demonstrations of transgenic modifications [13-15], suggest that genetic improvements of switchgrass through transgene expression and down-regulation of native genes will be accomplished with increasing regularity in the coming years.

Transformation of switchgrass and other monocots is facilitated by reliable molecular tools, including improved promoters for transgene expression. A variety of promoters used in monocot plant species have been reported in the literature, such as the rice (*Oryza sativa*) actin 1 (*OsAct1*) and actin 2 (*OsAct2*) promoters [16,17], the maize ubiquitin 1 (*ZmUbi1*) promoter [18], and multiple rice ubiquitin (*RUBQ1, RUBQ2, rubi3*) promoters [19,20]. However, relatively few promoters have been used in the production of transgenic switchgrass. Richards et al. [9] demonstrated that the *OsAct1*and *ZmUbi1 *promoters are able to drive transgene expression in switchgrass, and these promoters have been used in subsequent switchgrass transformation studies [11,21,22]. While the cauliflower mosaic virus (CaMV) *35S *promoter has been used in switchgrass, it typically resulted in lower levels of expression [21,23,24]. Somleva et al. [10] used the rice ubiquitin 2 (*rubi2*) promoter with limited success, whereas the *cab-m5 *light-inducible promoter from the chlorophyll *a/b*-binding protein in maize fused to the heat shock protein 70 (*hsp70*) intron resulted in significantly higher levels of transgene expression. However, *cab-m5 *expression is only expressed in a limited number of plant tissues and cell types [10,25,26]. Most recently, the *rubi3 *promoter was used to drive *sGFP *expression in switchgrass callus, stem, axillary bud and anther tissues [12,27]. Discovery and characterization of new promoters with enhanced levels of constitutive expression are needed [28], and would be highly beneficial to improve bioenergy feedstock crops through genetic transformation. While tissue-specific and inducible promoters are desirable for certain applications [29,30], constitutively expressed promoters are still the most commonly used promoters in transgenic plants and are advantageous for their wide range and stable levels of transgene expression.

Ubiquitin is a protein that consists of tandem repeats of a 76 amino acid monomer and is among the most conserved proteins in eukaryotes; only three amino acid polymorphisms exist among sequences from higher plant and animal species [31]. Ubiquitin is present in all eukaryotic cells. Therefore, the promoters from polyubiquitin genes are good candidates to regulate the constitutive expression of transgenes. Polyubiquitin gene promoters have been isolated from a variety of plants and tested for their ability to drive transgene expression, including from *Arabidopsis thaliana *[32], sunflower (*Helianthus annuus*) [33], parsley (*Petroselinum crispum*) [34], tobacco (*Nicotiana tabacum*) [35], potato (*Solanum tuberosum*) [36,37], tomato (*Solanum lycopersicum*) [38,39], sugarcane (*Saccharum oficinarum*) [40], *Gladiolus *sp. [41], soybean (*Glycine max*) [42] and *Lotus japonicus *[43] as well as the rice and maize polyubiquitin genes mentioned above. In contrast with the highly conserved nature of the protein-encoding portions of polyubiquitin genes, their promoters and introns have extensive sequence variability between paralogs and across organisms. However, all the polyubiquitin genes isolated from monocot and dicot plant species share similar structures, including a 5' UTR intron that significantly contributes to the strong expression capabilities of the polyubiquitin promoters [19,44-46].

In this study, we identified two novel polyubiquitin gene sequences (*PvUbi1 *and *PvUbi2*) from a switchgrass genomic library and characterized the native expression patterns of these genes. Additionally, reporter constructs were assembled containing the isolated 5' upstream regulatory regions of the coding sequences (i.e. *PvUbi1 *and *PvUbi2 *promoters) of these genes fused to the *uidA *coding region (*GUS*). These constructs were tested for transient and stable expression in a variety of plant species and tissues. Our results demonstrate the potential use of the *PvUbi1 *and *PvUbi2 *promoters in driving transgene expression in switchgrass, rice and tobacco. To the best of our knowledge, this is the first report characterizing native switchgrass promoter sequences for transgene expression.

## Results

### Sequence analysis of *PvUbi1 *and *PvUbi2*

**Figure 1:**
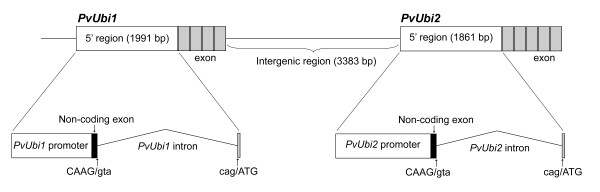
**Map of the *PvUbi1 *and *PvUbi2 *genes within the contiguous 10.4 kb sequence of switchgrass DNA in Pv9G7B5**. The light gray boxes represent the 228 bp ubiquitin monomer repeats of the translated exon. The black boxes represent the non-coding exons downstream of each TATA box for *PvUbi1 *and *PvUbi2*. The intron splices sites (CAAG/gta and cag/ATG) are shown.

Two polyubiquitin genes were cloned from a switchgrass fosmid library constructed by J. Hawkins and R. Percifield (unpublished results). These two genes were designated as ubiquitin 1 (*PvUbi1*) and 2 (*PvUbi2*) and are closely linked to each other within the genome (Figure 1). *PvUbi1 *consists of 607 bp containing *cis*-acting regulatory elements, a 5' untranslated region (UTR) containing a 93 bp non-coding exon and a 1291 bp intron, a 918 bp open reading frame (ORF) and a 191 bp 3' UTR. *PvUbi2 *consists of 692 bp containing *cis*-acting regulatory elements, a 97 bp 5' UTR, a 1072 bp intron, a 1146 bp ORF and a 183 bp 3' UTR. For *PvUbi1*, the 918 bp ORF encodes four tandem, head-to-tail repeats of 228 bp, commonly referred to as ubiquitin monomer repeats, with minimal sequence variation from one repeat to another. Similar results were found from sequence analysis of *PvUbi2*. However, instead of four repeats, the ORF contained five tandem head-to-tail repeats resulting in a coding region of 1146 bp. The ubiquitin monomers of *PvUbi1 *and *PvUbi2 *contained identical amino acid sequences compared to each other and several other plant species, including maize [18], Arabidopsis [47] and rice [19,20]. To test for promoter activity, the promoter candidate region of *PvUbi1 *that spans 607 bp of the 5' region upstream from the transcriptional initiation site, along with the 93 bp 5' UTR non-coding exon and the 1291 bp 5' UTR intron was cloned, resulting in a fragment of a total of 1991 bp. For *PvUbi2*, the isolated candidate promoter region was 1861 bp, including 692 bp upstream of the transcriptional initiation site, the 5' UTR exon (97 bp) and the 1072 bp 5' UTR intron (Additional file 1 Figure S1).

By analysis of the genomic DNA in the selected promoter region, introns were identified immediately upstream of the ATG start codon in the *PvUbi1 *and *PvUbi2 *genes. These introns were identified based on the consensus sequences CAAG/gtac at the 5' splice site and cag/ATG at the 3' splice site (Additional file 1 Figure S2), which are identical for characterized polyubiquitin genes from plants [18-20,33-36,40,45]. To validate these intron splice sites and identify the transcriptional initiation site, switchgrass mRNA was subjected to RACE-PCR with primers specific for *PvUbi1 *or *PvUbi2*. For *PvUbi1*, results revealed a transcriptional initiation site at an adenine located 1384 bases upstream from the ATG translational initiation codon of the polyubiquitin gene. Subsequently, the transcriptional initiation site for *PvUbi2 *was identified at an adenine 1169 bp upstream from the translational initiation codon of the polyubiquitin gene. Using cDNA clones from the RACE-PCR, along with expressed sequence tags (ESTs) from GenBank and Tobias et al. [48], the intron splice sites were confirmed, revealing 1291 and 1072 bp introns present in *PvUbi1 *and *PvUbi2*, respectively. The *PvUbi1 *and *PvUbi2 *introns exhibited limited homology (53%), similar to the level of identity that has been reported when comparing different rice ubiquitin introns [20].

The PlantCARE Database [49] was queried for putative *cis*-element sequences within the candidate promoter regions of *PvUbi1 *and *PvUbi2*. Several motifs of putative functionality were identified for *PvUbi1 *and *PvUbi2 *(underlined in Additional file 1, Figure S2). For *PvUbi1*, these consisted of motifs involved in anaerobic induction (TGGTTT, positions -577 to -572), light-responsiveness (ATTAATTTTACA, positions -350 to -339; CACGTC, positions -589 to -584; CC(G/A)CCC, positions -221 to 216; -170 to -165; and -54 to -49), response to methyl jasmonate (MeJA) (CGTCA, positions -392 to -388; -77 to -73), low-temperature responsiveness (CCGAAA, positions -112 to -107), endosperm expression (GTCAT, positions -391 to -387), a MYB transcription factor binding site involved in drought-inducibility (TAACTG, positions -312 to -307), three CAAT boxes (positions -553 to -550; -538 to -535; and -458 to -455) and a TATA box (TATATAAA, positions -33 to -26). For *PvUbi2*, the identified motifs for putative *cis*-acting regulatory elements were those involved in meristem expression (GCCACT) and meristem specific activation (CCGTCC), anoxic-specific inducibility (CCCCCG), low-temperature responsiveness (CCGAAA), endosperm expression (GTCAT), light responsiveness (CC(G/A)CCC), two CAAT boxes and a TATA box (TAAATA, positions -32 to -27). However, it is important to indicate that these elements were determined from *in silico *data and remain to be functionally validated.

### Tissue expression profiles of *PvUbi1 *and *PvUbi2*

**Figure 2:**
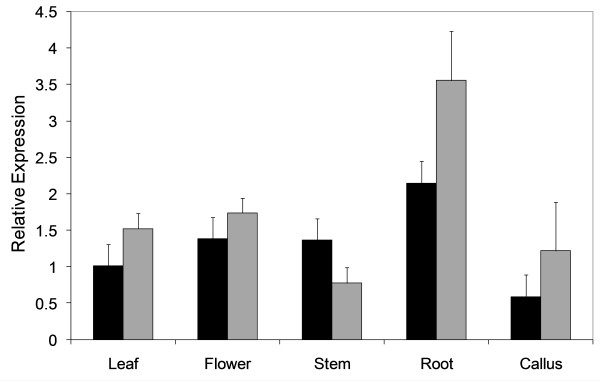
**Expression analysis of switchgrass *PvUbi1 *(black) and *PvUbi2 *(gray) in a variety of switchgrass tissues using qRT-PCR**. Relative quantification was performed using the standard curve method, and transcript accumulation of each gene was normalized to the quantity of an expressed switchgrass actin (*PvAct*) gene. Each bar represents the mean of three independent replicates with the standard errors of the noted mean.

A survey of switchgrass ESTs from the GenBank and Tobias et al. [48] databases revealed expression of *PvUbi1 *and *PvUbi2 *in all examined tissue types and growth stages: leaf, root, apex and stem, crown, callus, early floral buds and reproductive tissue, late flowering buds and seed development, and etiolated seedlings. To confirm these *in silico *data, specific primers were designed for *PvUbi1 *and *PvUbi2 *to perform quantitative reverse transcriptase-PCR (qRT-PCR) in different switchgrass tissues (Figure 2). The qRT-PCR results confirm that both *PvUbi1 *and *PvUbi2 *are expressed in all tissues tested (leaf, flower, stem, root and callus). *PvUbi2 *showed higher levels of expression in all tissues except stem when compared to *PvUbi1*.

### Transient and stable GUS expression regulated by the *PvUbi1 *and *PvUbi2 *promoters

The capabilities of the *PvUbi1 *and *PvUbi2 *promoter candidate regions to drive transgene expression were evaluated through the construction of expression vectors (Additional file 1, Figure S2). All promoter variants were cloned upstream of the *uidA *coding region (*GUS*) to create promoter-*GUS *fusions. Vector constructs were transformed into switchgrass callus by particle bombardment, and transient expression was observed by GUS histochemical staining. All constructs showed *GUS *expression, verifying that the *PvUbi1 *and *PvUbi2 *promoters can be used successfully to drive transgene expression. In order to compare expression levels of the *PvUbi1 *and *PvUbi2 *promoters with those of other promoters, several plant promoters commonly used in monocot transformation were selected and cloned into the same identical vector (pHLucGWgus) to eliminate any discrepancies in expression levels as a result of vector backbone or vector size (Additional file 1, Figure S2).

**Figure 3:**
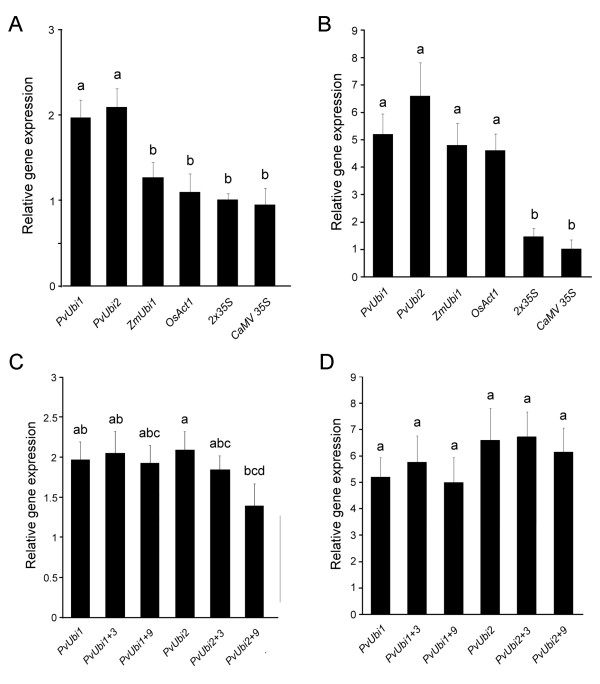
**Comparison of the relative levels of *GUS *under the control of different promoters in switchgrass (A, C) and rice (B, D) callus cultures**. Each promoter construct contained the same pHLucGWgus DNA backbone and the relative expression levels of *GUS *under the control of CaMV *35S *were set to 1 for both switchgrass and rice. All other promoter values are shown relative to this CaMV *35S *control. Bars represent the mean value of six independent replicates. Treatments that share the same letter are not significantly different as calculated by LSD (P ≤ 0.05). Error bars represent standard error.

**Figure 4:**
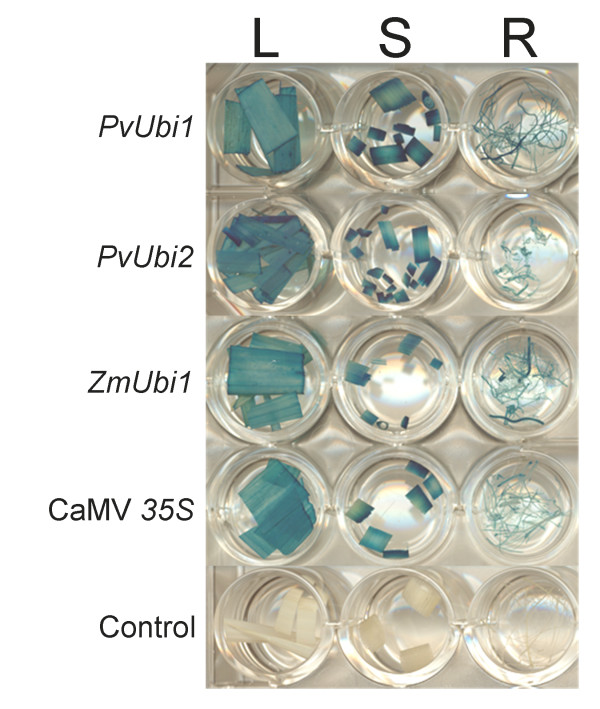
**Histochemical staining of GUS in rice leaves (L), stems (S) and roots (R) driven by different promoters**. The negative control is untransformed rice.

In switchgrass and rice, the *PvUbi1 *and *PvUbi2 *promoters resulted in higher levels of GUS compared to the CaMV *35S *and *2x35S *promoters (Figure 3). In switchgrass, the *PvUbi1 *and *PvUbi2 *promoters drove significantly higher *GUS *expression when compared side-by-side with all other plant promoters (*ZmUbi1, OsAct1, 2x35S*, CaMV *35S*), with the *PvUbi2 *promoter reaching 2.1-fold higher levels than CaMV *35S *(Figure 3a). In rice, the *PvUbi1 *and *PvUbi2 *promoters resulted in 5.1- and 6.6-fold higher levels of expression when compared to CaMV *35S*, respectively (Figure 3b). No enhancement of GUS activity was observed when three and nine amino acids of the *PvUbi1 *and *PvUbi2 *ubiquitin coding regions were fused to their respective promoters (Figure 3c and 3d). When comparing promoter expression levels between switchgrass and rice, the relative difference in absolute gene expression was 2.6- to 4.4-fold higher for rice. To further validate these transient assays, rice callus was bombarded with promoter- pHLucGWgus constructs and selected for stable transformation. When transgenic plants were grown to maturity, the *PvUbi1 *and *PvUbi2 *promoters produced GUS in leaves, stems and roots of mature rice plants, while no GUS was detected in the untransformed control (Figure 4).

**Figure 5:**
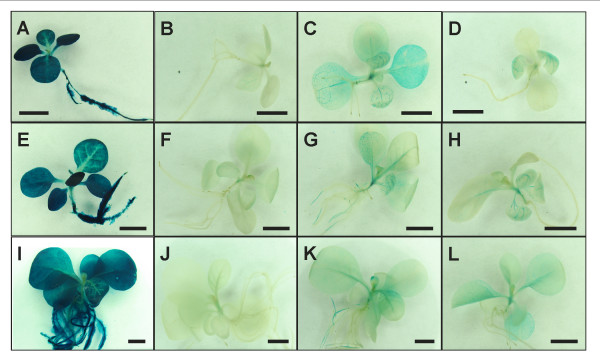
**Histochemical staining of GUS in tobacco seedlings driven by the *2x35S *(A, E, I), *PvUbi2 *(B, F, J), *PvUbi2+3 *(C, G, K) and *PvUbi2+9 *(D, H, L) promoters**. Staining was performed at 10 days (A, B, C, D) and 17 days (E, F, G, H) post-germination. Heat shock was also performed at 17 days post-germination (I, J, K, L). Each measurement bar represents 5 mm.

The *PvUbi1 *and *PvUbi2 *promoter constructs were stably transformed into tobacco (cv. Xanthi) to evaluate expression levels of these promoters in a dicot expression system. Additional promoter variants containing three or nine amino acid fusions downstream of the *PvUbi1 *and *PvUbi2 *intron sequences were tested for the potential of increased transgene expression. Stably transformed T_0 _plants were randomly selected and grown to obtain seed for generation of T_1 _progeny. GUS from the *PvUbi1, PvUbi1+3 *and *PvUbi1+9 *promoter constructs could not be visually observed in T_1 _seedlings at 10 and 17 days after germination, so these promoter variants were not studied in subsequent experiments (data not shown). While the *PvUbi2 *promoter drove *GUS *expression in leaves, stems and roots at 10 and 17 days, the levels of expression were minimal when compared to *2x35S *(Figure 5). However, the *GUS *expression dramatically increased with the *PvUbi2 *promoters containing an additional three and nine amino acid fusions from the ubiquitin coding region (*PvUbi2+3 *and *PvUbi2+9*), exhibiting visibly detectable levels of GUS activity in the vascular tissue of leaves, stems, and roots. There was no visible enhancement of GUS from the *PvUbi2 *promoter constructs in tobacco following heat shock induction treatment for 60 minutes at 42°C. However, heat shock induction was not experimentally tested within the native *PvUbi1 *or *PvUbi2 *genes in switchgrass.

**Figure 6:**
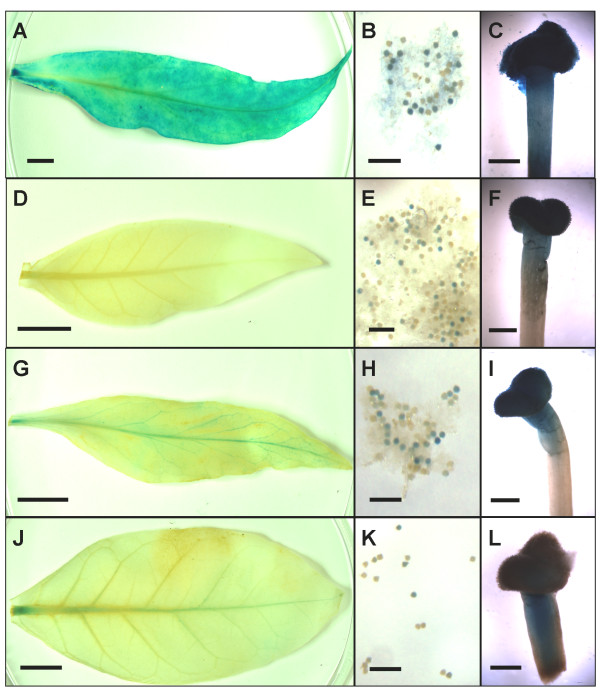
**Histochemical staining of GUS in the leaves (A, D, G, J), pollen (B, E, H, K) and pistils (C, F, I, L) of mature tobacco plants driven by the *2x35S *(A, B, C), *PvUbi2 *(D, E, F), *PvUbi2+3 *(G, H, I) and *PvUbi2+9 *(J, K, L) promoters**. Each measurement bar represents 1 cm (leaf), 0.2 mm (pollen) and 1 mm (pistil).

Since the *PvUbi2 *promoters showed expression in seedlings, plants containing these promoter constructs were grown to maturity for further analysis. As shown in Figure 6, adult T_1 _plants showed staining of GUS in the pollen, pistil and leaves for the *PvUbi2 *promoter variants (*PvUbi2, PvUbi2+3, PvUbi2+9*). Similar to the data from young seedlings, the *PvUbi2+3 *and *PvUbi2+9 *promoters containing fusions showed the highest levels of expression. Expression of *GUS *in mature leaves under the control of *PvUbi2 *promoter variants appeared to be specific to the vascular tissue. *GUS *expression driven by the *PvUbi2+3 *and *PvUbi2+9 *promoters in mature T_1 _plants was observed to be lower than those in seedlings. GUS staining was consistently higher for the *2x35S *promoter in both tobacco seedlings and mature adult T_1 _plants.

## Discussion

This study demonstrates the isolation and characterization of two switchgrass polyubiquitin genes, *PvUbi1 *and *PvUbi2*. The identical amino acid sequences of the ubiquitin monomer repeats in *PvUbi1 *and *PvUbi2 *further reveal the highly conserved nature of the polyubiquitin gene family across both monocot and dicot genes [19,20,32,35,37,40]. Both *PvUbi1 *and *PvUbi2 *displayed high levels of native expression in all tissue types analyzed, consistent with the broad function of ubiquitin in cell-cycle regulation [50], DNA-repair [51] and other processes required of all cell types [31]. Additional sequence data (unpublished) suggest that more than one copy of polyubiquitin genes could exist in switchgrass containing 5' and 3' UTR regions identical to that of *PvUbi1*. These data are not unique to this study, as similar observations have been made in sugarcane, where 5' and 3' UTR sequences were identical for two individually isolated clones, while the number of ubiquitin monomer coding repeats varied [40]. Whether these findings are the results of different polyubiquitin genes present in the switchgrass genome or of post-transcriptional splicing and modification remains to be determined. In either case, the presence of multiple genes is consistent with the polyploidy nature of these species.

Intron-mediated enhancement of gene expression has been shown in a number of plant species [44,52], and multiple ubiquitin promoters have shown enhanced transgene expression when the intron is included in the promoter region [19,44-46,53,54]. Therefore, the intron regions (1291 bp for *PvUbi1 *and 1072 bp for *PvUbi2*) were retained with their respective 5' upstream promoter candidate regions during vector construction. However, the regulatory elements of some monocot promoters retain high expression despite large deletions in the internal portions of the intron sequence, as long as efficient intron splicing is retained [16]. Therefore, future deletion analysis for the *PvUbi1 *and *PvUbi2 *promoter candidate regions could yield beneficial results, since the removal of internal ubiquitin intron sequences may have no detrimental effect [35].

The fusion of ubiquitin monomers to the N terminus of expressed proteins has been observed to lead to site-specific and highly efficient cleavage, resulting in free ubiquitin and free protein of interest in the cell [55]. These fusions are not only efficiently processed and cleaved by ubiquitin-specific proteases, but can also result in enhanced gene expression and protein accumulation [19,56]. Ubiquitin fusions have been applied for higher production of therapeutic recombinant proteins [57]. Additionally, plant vectors have been developed that employ ubiquitin fusions for the coexpression and cleaving of two proteins from a single transcript [58]. This approach has also been used for the enhancement of ubiquitin promoters in regulating transgene expression by the addition of an entire ubiquitin monomer or select amino acids from the N terminus of the polyubiquitin coding region downstream of the promoter region [19,36,37,46]. In this study, comparative expression levels of the *PvUbi1 *and *PvUbi2 *promoter candidate regions and their three (*PvUbi1+3 *and *PvUbi2+3*) or nine (*PvUbi1+9 *and *PvUbi2+9*) amino acid ubiquitin fusion promoter variants revealed minimal changes in levels of *GUS *expression for switchgrass and rice. However, the three and nine amino acid fusions of the polyubiquitin coding region to the *PvUbi2 *promoter and intron drastically increased GUS staining in the vascular tissue of tobacco. While an additive effect has been observed when the first three or nine amino acids of the polyubiquitin coding region are fused to the N-terminus of a transgene and coupled with a ubiquitin promoter [19], these enhancing effects were later attributed to the removal of a *GUS *5' untranslated leader sequence [59]. Therefore, the enhancing effect of these fusions in tobacco is striking, since the majority of ubiquitin fusion enhancements have been demonstrated fusing an entire ubiquitin monomer to the transgene [36,37,46,56,60]. Fusion of the entire ubiquitin monomer was not tested in this study, but the high levels of expression in switchgrass and rice using the *PvUbi1 *and *PvUbi2 *promoters and introns without the ubiquitin monomer fusions demonstrates that these promoter and intron regions alone are highly useful for plant biotechnology applications in monocots. Likewise, the *PvUbi2+3 *and *PvUbi2+9 *promoter variants could be useful for tissue-specific applications in tobacco and other dicots.

Data from transient biolistic bombardment assays can be highly variable in nature. Therefore, the rice and switchgrass transient expression assays were repeated in six independent replicates to increase the reliability of the resulting data. In addition, the pHLucGWgus vector used in these experiments contained the *ZmUbi1 *promoter and the firefly luciferase coding sequence (*LUC*) in the vector backbone as an internal control to further improve the reliability and reproducibility of these transient expression data, as previously reported [19,61,62]. The methodology used for the bombardment assay in switchgrass and rice is the first plant transformation study of promoter expression using an internal *LUC *cassette control within the same vector backbone as the experimental promoter-*GUS *fusion cassette, as opposed to the standard use of co-transformation [19,63,64].

The promoter-*GUS *fusions were observed to produce high levels of transgene expression in both switchgrass and rice. The comparisons of the expression levels of promoters *PvUbi1 *and *PvUbi2 *to other promoters are worth noting, since the CaMV *35S *and *ZmUbi1 *promoters are the two most commonly used in monocot transformation. The *PvUbi1 *and *PvUbi2 *promoters showed levels of expression higher than or equal to those of the *ZmUbi1 *and CaMV *35S *promoters. The *PvUbi2 *promoter should be the most effective for driving expression of transgenes in switchgrass; promoter *PvUbi2 *resulted in significantly higher expression levels than *ZmUbi1 *and CaMV *35S*. In rice, the differences were even more striking, with the *PvUbi2 *promoter resulting in expression levels 6.6-fold higher than that of CaMV *35S*, the most commonly used promoter in this species. The levels of gene expression detected in rice for the *ZmUbi1, OsAct1 *and CaMV *35S *promoters in this study reflect previous findings of high gene expression levels in maize [62]. The *PvUbi1 *and *PvUbi2 *promoters resulted in higher transgene expression in rice than in switchgrass, demonstrating the flexibility of these promoters in a monocot species other than switchgrass, and the potential for similar results in other monocots as well. The stable expression of *GUS *driven by *PvUbi1 *and *PvUbi2 *promoters in rice leaves, stems and roots also demonstrates the ubiquitous nature of these promoters in mature plants. These data suggest that the *PvUbi1 *and *PvUbi2 *promoters should be very effective for driving constitutive transgene expression in switchgrass, rice and potentially other monocots.

While expression of the *PvUbi1 *promoter was not observed in tobacco, the tissue-specific regulation of the *PvUbi2 *promoter and fusion variants could make these promoters advantageous for some applications that require expression limited to vascular or reproductive tissues. The decrease of GUS expression in adult leaf tissue is consistent with the findings that ubiquitin levels are higher in younger plant tissues [47,65], although conflicting observations have been made with other ubiquitin promoters [66]. When the *PvUbi2 *promoter variants were tested for heat shock-induced transgene expression in tobacco, there was no effect observed on the level of *GUS *expression. However, it is worth noting that the *ubq1-1 *promoter from tomato exhibited increased native transcript levels under heat shock conditions, but once fused to the *GUS *gene and stably expressed in tobacco, exhibited no response to heat shock induction [39], suggesting that the heat shock of ubiquitin promoters can yield different results when compared between native species and transgenic plant expression. In contrast, heat shock induction of the *ZmUbi1 *promoter resulted in increased expression levels in native maize tissue as well as transgenic rice callus and protoplasts [18,65,67], and sugarcane [40]. However, heat shock has been reported to have a variety of effects on polyubiquitin promoters including up-regulation [18-20,33], down-regulation [38,68] and no change [34]. The heat shock element consensus sequence (CNNGAANNTTCNNG) reported by Pelham [69] was not found in the promoter regions of *PvUbi1 *or *PvUbi2*, and no heat shock elements were identified from the PlantCARE database queries [49]. However, the numerous putative *cis*-acting regulatory elements identified in the promoter regions of *PvUbi1 *and *PvUbi2 *could be functionally validated in future studies.

## Conclusion

Because ubiquitin is a necessary component of all eukaryotic cells, polyubiquitin genes are prime candidates for the isolation of highly expressed constitutive promoters. We identified and characterized promoters from two polyubiquitin genes (*PvUbi1 *and *PvUbi2*) in switchgrass. Experiments using these promoters resulted in high levels of expression in switchgrass and rice that equaled or surpassed all of the commonly used plant promoters tested in this study (*ZmUbi1, OsAct1*, CaMV *35S *and *2x35S*). In addition, stable transformation of tobacco with the *PvUbi2+3 *and *PvUbi2+9 *promoter fusion variants showed expression in seedlings as well as the leaves, pistils and pollen of mature plants. These data suggest that the *PvUbi1 *and *PvUbi2 *promoters are valuable for genetic transformation studies and demonstrate the potential broad versatility of these promoters in monocot and dicot species.

## Methods

### Construction and screening of a switchgrass fosmid library

*Panicum virgatum *(cv. Alamo) leaf tissue was used to construct a fosmid library (unpublished data). In order to screen the library for polyubiquitin genes, *P. virgatum *EST sequence data (from JGI, Walnut Cove, CA) were aligned to genomic DNA sequences from rice and maize (NCBI). From these alignments, primers were designed to amplify fragments of 700-760 bp in size. Sequence specific primers (5'-TBACYGGMAAGACBATHACY-3', 5'-TCCTTYTGRATGTTRTARTC-3') were then used to screen the library. Fosmids identified to contain polyubiquitin genes were grown in 50-ml cultures containing 50 μl of fosmid induction solution (Epicentre Biotechnologies) to increase copy number, as per manufacturer's protocol. Nuclear-free fosmid DNA was extracted using the Qiagen Large Construct Kit. Approximately 10 μg of fosmid DNA [66 ng μl^-1^] was sheared to 2-10 kb using the Standard Hydroshear Shearing Assembly (Genomic Solutions) for 20 cycles at a speed code of 16. Sheared fragments between 3 - 8 kb were excised and shotgun libraries were built as described [70]. Ten clones were randomly picked from the sub-clone library and digested with *Eco*RI to determine the average insert size as quality control. A total of 384 sub-clones were sequenced from both directions using ABI PRISM BigDye Chemistry (Applied Biosystems, Foster, CA) and run on an ABI 3730. The sequences were assembled using Phred/Phrap and annotated in Apollo [71]. The cumulative data represent an approximate 13-fold coverage of each fosmid. Fosmid Pv9G7B5 contained two polyubiquitin genes in tandem and in the same orientation, both of which showed ≥ 99.5% sequence identity to switchgrass ESTs from callus, early floral development, late floral development, root, and stem tissues. Contig Pv9G7B5 was used for further isolation of switchgrass ubiquitin promoters.

### Sequence analysis

Predictions were made for the location of the ubiquitin promoters and genes within the Pv9G7B5 fosmid using FGENESH [72] and GENSCAN [73] and further confirmed using blastn in GenBank and aligned with homologous ubiquitin sequences from other plant species in AlignX (Invitrogen, Carlsbad, CA). Based on these results, primers were designed to produce amplicons of *PvUbi1 *(1991 bp) and *PvUbi2 *(1861 bp) upstream of the predicted transcription start site (Additional file 1, Table S1). Identification of putative regulatory *cis*-elements within the promoter regions of *PvUbi1 *and *PvUbi2 *was performed using the PlantCARE database (http://bioinformatics.psb.ugent.be/webtools/plantcare/html) [49].

### RACE-PCR

The full-length cDNAs (including 5'UTRs, coding sequence and 3'UTRs) of the *PvUbi1 *and *PvUbi2 *genes were identified using the 5'RACE-PCR and 3'RACE-PCR, respectively, in the GeneRacer™ kit (Invitrogen, Carlsbad, CA, USA). Total RNA extractions from leaves of switchgrass *cv*. Alamo were performed using the TRI reagent (MRC, Cincinnati, OH). Approximately 3 μg of total RNA were used for reverse transcription to generate cDNA. To remove trace contamination of genomic DNA, RNA was treated with DNase I according to manufacturer's instructions (Promega, Madison, Wisconsin, USA). The resulting 5' and 3'UTRs of cDNA of both genes were amplified with the GeneRacer™ kit and cloned into pCR^®^8/GW/TOPO^® ^vector (Invitrogen) for sequence confirmation and analysis. The primers are listed in Additional file 1, Table S1.

### Quantitative reverse transcriptase PCR (qRT-PCR)

Levels of *PvUbi1 *and *PvUbi2 *mRNA abundance were measured using quantitative reverse transcriptase PCR (qRT-PCR) in a variety of switchgrass tissues. Flower, leaf, stem and root tissues of three-month-old greenhouse-grown switchgrass (cv. Alamo), and callus generated from inflorescences of a switchgrass genotype (Alamo 2) [8] were used for RNA extraction. Total RNA was isolated using Tri-Reagent (Molecular Research Center, Cincinnati, OH), and DNA contamination was removed with DNase treatment (Promega, Madison, WI) following the manufacturer's instructions. A switchgrass actin gene (*PvAct*) was used as an internal control. Specific primers to the corresponding genes were designed (Additional file 1, Table S1) that amplify a single product for each corresponding gene, as confirmed by the melting temperature of the amplicons and gel electrophoresis. Approximately 3 μg of the total RNA from three independent experiments were synthesized into first strand cDNA using the High Capacity cDNA Reverse Transcription kit (Applied Biosystems, Foster City, CA) and qRT-PCR was conducted in triplicate using Power SYBR Green PCR master mix (Applied Biosystems) according to the manufacturer's protocol. Relative quantification was performed using the standard curve method, and transcript accumulation of each gene was normalized to the quantity of expressed switchgrass actin gene. For quality assurance purposes, only qRT-PCR assays that resulted in standard curves with the following parameters were considered: 1) linear standard curve throughout the measured area, 2) standard curve slope between -3.5 and -3.2, and 3) R^2 ^value above 0.99.

### Expression vector construction

All promoters (*ZmUbi1, OsAct1*, CaMV *35S, 2x35S, PvUbi1, PvUbi1+3, PvUbi1+9, PvUbi2, PvUbi2+3, PvUbi2+9*) were amplified with specific primer sets shown in Additional file 1 (Table S1) and cloned into pCR8/GW/TOPO (Invitrogen, Carlsbad, CA). The *PvUbi1 *and *PvUbi2 *promoter variants were derived from the Pv9G7B5 contig mentioned above, the *ZmUbi1 *promoter from pAHC25 [74], the *OsAct1 *promoter from pCOR113 [75], the CaMV *35S *promoter from pBin-m-gfp5-ER [76], and the *2x35S *promoter from pMDC32 [77]. DNA was confirmed by restriction enzyme digests for orientation, and clones containing the proper orientation were sequence-verified at the University of Tennessee Molecular Biology Resource Facility. These amplified promoter regions were introduced from the pCR8/GW/TOPO backbone into the binary vectors pGWB533 and pGWB535 [78] using the Gateway^® ^LR Clonase^® ^II enzyme mix (Invitrogen). The pGWB533 vector contains the Gateway^® ^cassette upstream of the *uidA *coding region (*GUS*), resulting in promoter:*GUS *fusion constructs used for initial promoter analysis and tobacco transformations. For comparison of different promoters in switchgrass and rice, the pCR8/GW/*ZmUbi1 *vector and the pGWB535 vector (containing the Gateway^® ^cassette upstream of the firefly luciferase coding region (*LUC*)) were LR recombined and the resulting *ZmUbi1*:*LUC *cassette was cloned along with the Gateway^® ^cassette upstream of *GUS *(cloned from pGWB533) and termed pHLucGWgus. The reporter gene cassettes were assembled, sequenced and annotated using Geneious v5.0.3 software [79]. Each unique promoter described above was LR recombined into the Gateway-compatible site of the pHLucGWgus vector upstream and in the correct frame for GUS protein synthesis and sequence verified.

### Plant materials and tissue culture

Switchgrass *cv*. Alamo genotype ST1 was provided by Zeng-Yu Wang from the Noble Foundation [80]. Plants were maintained in the greenhouse by pruning tillers that matured beyond the boot stage [81] in a 42% sand, and 58% Fafard 3B soil mix (Conrad Fafard, Inc., Agawama, MA) in 12-liter plastic pots. Growth conditions consisted of a 12-h light/12-h dark cycle under 400-watt halide lamps. The greenhouse temperatures ranged from 20-27°C. Plants were watered daily, and fertilized weekly with 0.45 kg of Peters^® ^Professional All Purpose Plant Food (St. Louis, MO) per 379 liters of water. The last culm node of switchgrass produced immature inflorescences at the E2-R0 stages [81]. Culm nodes were identified as previously described by Alexandrova et al. [82]. The 6.5-cm explants were surface-sterilized with 70% EtOH for 1 minute with gentle agitation. Explants were then placed in 15% Clorox^® ^v/v supplemented with 0.01% Tween-20 (Fisher Scientific, Pittsburgh, PA, USA) and gently agitated for 3 minutes. All tissues were then rinsed three times at 2-minute intervals. Sterilized internodes were cut in half longitudinally [82] and explants were placed cut-side down on solid Murashige and Skoog (MS) medium [83] supplemented with B5 vitamins, 5 μM BAP and 3% sucrose. Explants were incubated in a growth chamber at 25°C, with cool-white fluorescent lighting (66-95 μE m^-2 ^s^-1^) 16-h light/8-h dark cycle for 14 days. After 14 days of culture, immature inflorescences were used to initiate embryogenic callus cultures. The inflorescences were dissected out and cut to obtain sections of rachis tissue measuring 1 cm in length. Inflorescence pieces were placed on solid N6E medium [84] and incubated at 27°C in the dark with subculturing at three-week intervals. After the second subculture, callus was separated from the inflorescences and arranged in a 5 × 5-grid pattern on plates. Friable embryogenic callus tissue was bulked for eight months with subcultures at three-week intervals and used in particle bombardment experiments.

Seeds of rice *cv*. Taipei 309 were provided by the USDA National Plant Germplasm System. Kernels from dehusked seeds were surface-sterilized in 70% EtOH for 2 minutes at 100 RPM. Kernels were then transferred to a 60% Clorox^® ^v/v supplemented with 0.01% Tween-20, stirred for 30 minutes and rinsed three times with H_2_O for two minutes. Sterilized kernels were dried, arranged in a 5 × 5-grid on modified NB medium (MNB) as per Chen et al. [85] and incubated in the dark at 27°C. Prior to particle bombardment, rice callus was induced, selected, and maintained as previously described for 5 months with transfers at 3-week intervals [85]. All switchgrass and rice media were solidified with 2.5 g l^-1 ^Gelzan™ (Caisson Laboratories, North Logan, UT, USA) and brought to pH 5.8 prior to autoclaving. Cultures were sealed in Petri dishes with 3M Micropore™ tape (St. Paul, MN, USA).

### DNA particle bombardment of switchgrass and rice callus

Transient expression assays of Taipei 309 and ST1 embryogenic callus cultures were conducted following biolistic transformation using the Bio-Rad PDS-1000 (Bio-Rad Laboratories, Hercules, CA). The PDS-1000 was used for plasmid delivery with 7,584 kPa (1,100 psi) rupture disks, a microcarrier flight distance of 6 cm and a vacuum of 97 kPa (27 in) Hg [86,87], with all hardware and reagents produced by Bio-Rad. Microprojectile preparation essentially followed Trick et al. [88] with the DNA amount decreased from 625 ng to 300 ng per bombardment, and 10 mg of 0.6 μm diameter gold (Au) particles used instead of 12 mg of 1 μm particles. Each bombardment consisted of a 10 μl aliquot placed on the macrocarrier and allowed to dry completely. Switchgrass and rice callus cultures were incubated for 6 h prior to bombardment on N6 osmotic medium with 0.6 M osmoticum (http://www.agron.iastate.edu/ptf/protocol/Callus%20bb.pdf), or 0.6 M NB osmotic medium [85], respectively. Each vector was used to bombard six replicate plates with 50 callus pieces per plate. To test the functionality of the promoter vectors and the validity of the bombardment assay, ten rice calli were selected from each of the first two replications and histochemically stained for observation of GUS. The five rice calli with the highest level of expression were selected and photographed (Additional file 1, Figure S3).

### Stable transformation of rice

Stable transformations of rice were performed as described above for transient expression assays with three exceptions: three-month-old rice callus cultures were used and 150 ng of the pHLucGWgus vectors (containing the *PvUbi1, PvUbi1+3, PvUbi1+9, PvUbi2, PvUbi2+3, PvUbi2+9, ZmUbi1*, and CaMV *35S *promoters) were used per bombardment. Rice callus cultures were incubated for 6 h pre- and 18 h post-bombardment on 0.6 MNB osmotic medium [85]. Rice callus cultures were selected on MNBH50 as described [85] to ensure independent events were recovered. Positive transgenic calli were regenerated as described by Broothaerts et al. [89] on RGH6 medium solidified with Phytagel (6 g l^-1^) without selection and resulting plantlets were rooted for four weeks on 1/2 MS medium supplemented with B5 vitamins and hygromycin B (50 mg l^-1^) solidified with 3 g l^-1 ^Gelzan™ in Magenta^® ^GA-7 Plant Culture vessels. Regeneration and rooting occurred under a 23-h light/1-hr dark photoperiod provided by cool-white fluorescent light (66-95 μE m^-2^s^-1^) at 26°C. Prior to being moved to the greenhouse, a root sample was harvested and GUS-stained for all transgenics, and an untransformed control was regenerated without selection. Plants were allowed to grow in the greenhouse for approximately two months prior to harvesting tissue for GUS staining of leaf and stem tissues.

### MUG and LUC assays

Following bombardment, gene expression was analyzed using luciferase and MUG assays. Thirty-six hours post-bombardment, 25 calli per replicate were ground in 50 μl of 1× lysis buffer (1× LB) [63]. For the first two replications, five calli were stained for GUS [90]. Upon lysing the cells, 350 μl of additional 1× LB were added to each sample. The cell lysates were centrifuged at 13,000 g for five minutes at ambient conditions; the tubes were then rotated 180° and spun again. The soluble protein extracts produced from each sample were used for 4-methylumbelliferyl β-D-glucuronide (MUG) and luciferase assays [63,64]. For MUG assays, 50-μl of protein extract were added to 50 μl of assay buffer (1 mM MUG in 1× LB). Reactions were incubated at 37°C for 24 hours, and subsequently terminated with 100 μl of stop buffer (0.2 M Na_2_CO_3 _in H_2_O). Samples were read in duplicate with the BioTek^® ^Synergy 2 fluorometer (BioTek, Winooski, VT, USA) at an excitation wavelength of 360/40 nm and an emission wavelength of 460/40 nm. The fluorometer was calibrated with 4-methyl umbelliferone (MU) standards in stop buffer. MUG results were expressed as micromole MU released hour^-1^. Luciferase activity was quantified twice for each replicate using 25 μl of protein extract. For each sample reading, 25 μl of sample extract in 1× LB buffer were diluted in 75 μl of Glo-lysis buffer, mixed with 100 μl of ONE-Glo™ Luciferase Assay buffer (Promega Corporation, Madison, WI, USA) and allowed to incubate at room temperature for 5 minutes. Non-specific GUS and luciferase activity was corrected, and normalization of the MUG data was accomplished using luciferase activity as previously described [62]. Each unique *GUS *cassette allowed the measurement of gene expression to be quantified. The strength of each promoter was reported relative to that of the CaMV *35S *control, normalized to 1, to create a dimensionless value of promoter strength [19,62].

### Agrobacterium*-mediated transformation of tobacco*

The vectors to be tested were transformed into *A. tumefaciens *EHA105 as previously described [91]. EHA105 cells were maintained in liquid YEP medium and all incubations were performed at 28°C. Tobacco cv. Xanthi seeds were surface-sterilized, transformed, and regenerated using 50 mg l-1 hygromycin for selection according to published methods [92].

### Histochemical staining

Plant tissues were stained for GUS activity in microwell plates and placed at 37°C overnight as described [90]. For tobacco, intact tissue stains were made homogenous by vacuum infiltrating in solution for 30 minutes. For optimal visualization of stained tissues, chlorophyll was removed by repeatedly washing the tissue with a solution containing a 3:1 ratio of EtOH and acetic acid, ultimately storing tissue samples in 70% EtOH for imaging. For rice tissues, GUS staining assays were completed using a modified GUS buffer [93] brought to pH 7 [94], and chlorophyll was removed from the tissues as described by Cervera [95].

### Statistical analysis

Data for relative expression of promoters using the MUG and LUC assays were subjected to Levene's test [96] to check for homogeneity of variance using the software package JMP^® ^(Version 8.0.2 SAS Institute Inc., Cary, NC). When p ≤ 0.05, the data were considered to have unequal variances and were subjected to a square root transformation prior to ANOVA. Data sets with equal variances were subjected to ANOVA. If a significant difference was detected (p ≤ 0.05) using ANOVA, the least significant difference test (LSD) was employed to analyze the data for significant differences between treatments within an experiment (p = 0.05).

### GenBank accession numbers

The *PvUbi1 *and *PvUbi2 *genes containing the promoters, 5' UTR exons and introns, polyubiquitin ORFs, and the 3' UTR regions have been deposited in GenBank (accession numbers HM209467 and HM209468, respectively).

## Authors' contributions

DGJM designed and performed all experiments corresponding to the isolation and characterization of the individual gene elements and their respective promoters, the construction of vectors and the functional validation of the promoters in different species, analyzed the data and drafted the manuscript. ZRK designed and performed the promoter comparison assays in switchgrass and rice and assisted with revisions to the manuscript. WL designed and performed experiments of RACE-PCR and qRT-PCR. BLJ performed stable transformation in tobacco and assisted with revisions to the manuscript. RJP and JSH constructed and screened the fosmid library for the presence of polyubiquitin genes. PRL participated in the design and construction of vectors. BJA and JNB participated in particle bombardment experiments. MM participated in experimental design, data analysis and assisted with revisions to the manuscript. JLB, WAP and CNS conceived and coordinated the study, and assisted with revisions to the manuscript. All authors read and approved the final version of this manuscript.

## Supplementary Material

Additional file 1 **Supplemental data**.These data include sequences of the promoter candidate regions for *PvUbi1 *and *PvUbi2*, vector diagrams, representative images of the biolistic transformations and sequences of primers used in this study. Click here for file
