# Supplementary material for: Switchgrass (Panicum virgatum L.) polyubiquitin gene (PvUbi1 and PvUbi2) promoters for use in plant transformation
Source: BMC Biotechnol. 2011 Jul 11;11:74. doi: 10.1186/1472-6750-11-74 (PMC3161867; doi:10.1186/1472-6750-11-74)
Supplement: Additional file 1 — Supplemental data.These data include sequences of the promoter candidate regions for PvUbi1 and PvUbi2, vector diagrams, representative images of the biolistic transformations and sequences of primers used in this study. [file 1472-6750-11-74-S1.DOC]

Switchgrass (*Panicum virgatum* L.) polyubiquitin gene (*PvUbi1* and *PvUbi2*) promoters for use in plant transformation

David GJ Mann^1,4,*^, Zachary R King^2,4^, Wusheng Liu^1^, Blake L Joyce^1^, Ryan J Percifield^3,4^, Jennifer S Hawkins^3,4^, Peter R LaFayette^2,4^, Barbara J Artelt^2,4^, Jason N Burris^1,4^, Mitra Mazarei^1,4^, Jeffrey L Bennetzen^3,4^, Wayne A Parrott^2,4^, Charles N Stewart, Jr^1,4^

^1^Department of Plant Sciences, University of Tennessee, Knoxville, TN 37996, USA

^2^Department of Crop and Soil Sciences, University of Georgia, Athens, GA 30602, USA

^3^Department of Genetics, University of Georgia, Athens, GA 30602, USA

^4^The BioEnergy Science Center, Oak Ridge National Laboratory, Oak Ridge, TN 37831-6026, USA

^*^Corresponding author, email [dmann1@utk.edu](mailto:dmann1@utk.edu)

**SUPPLEMENTAL DATA**

**Supplementary Figure Legends**

Figure S1. The sequences of the promoter candidate regions for *PvUbi1* (A) and *PvUbi2* (B). These include potential regulatory elements (underlined). The putative TATA boxes are double underlined and the transcriptional initiation site is designated as +1. Uppercase letters represent the 5’ putative promoter region containing regulatory elements. Upper case italic letters represent the 5’ untranslated sequence of the non-coding exon. Lower case letters represent the intron sequence. Upper case italic bold letters represent the exon with the corresponding amino acid displayed beneath each codon. The forward slashes represent the boundary between the exon and intron with the intron splice sites shown in bold.

Figure S2. Schematic diagrams of the pHLucGWgus backbone used for biolistic bombardment in switchgrass and rice callus cultures. Various promoters recombined into the vector construct to drive *GUS* expression are shown in gray. Abbreviations: *PvUbi1* (switchgrass polyubiquitin 1 promoter), *PvUbi2* (switchgrass polyubiquitin 2 promoter), *ZmUbi1* (maize ubiquitin 1 promoter), *OsAct1* (rice actin 1 promoter), CaMV *35S* (cauliflower mosaic virus 35S promoter), *2x35S* (dual CaMV *35S* promoter), *GUS* (gene encoding for β-glucuronidase), *LUC* (gene encoding for luciferase), *hph* (hygromycin resistance gene), nos T (*A. tumefaciens nos* terminator sequence), 35S T (CaMV *35S* terminator), Kan^r^ (bacterial kanamycin resistance gene).

Figure S3. Representative images of rice calli after histochemical staining of GUS following biolistic transformation with no vector (A), or the pHLucGWgus construct containing the *PvUbi1* (B), *PvUbi2* (C)*, ZmUbi1* (D)*,* *OsAct1*(E), *2x35S* (E) and CaMV *35S* (K) promoters. (L) A schematic of the plate layout and the sampling distribution of calli selected for the visualization of GUS. Yellow circles represents the five calli selected per replicate plate that were bombarded for imaging. The scale bar represents 2mm.

A. *PvUbi1*

CCACTGGAGAGGGGCACACACGTCAGTGTTTGGTTTCCACTAGCACGAGTAGCGCAATCAGAAAATTTTCAATGCAT

-607 -589 -584 -577 -572 -553 -550 -538 -535

GAAGTACTAAACGAAGTTTATTTAGAAATTTTTTTAAGAAATGAGTGTAATTTTTTGCGACGAATTTAATGACAATA

-458 -455

ATTAATCGATGATTGCCTACAGTAATGCTACAGTAACCAACCTCTAATCATGCGTCGAATGCGTCATTAGATTCGTC

-392 -388

-391 -387

TCGCAAAATAGCACAAGAATTATGAAATTAATTTTACAAACTATTTTTATTTAATACTAATAATTAACTGTCAAAGT

-350 -339 -312 -307

TTGTGCTACTCGCAAGAGTAGCGCGAACCAAACACGGCCTGGAGGAGCACGGTAACGGCGTCGACAAACTAACGGCC

ACCACCCGCCAACGCAAAGGAGACGGATGAGAGTTGACTTCTTGACGGTTCTCCACCCCTCTGTCTCTCTGTCACTG

-221 -216 -170 -165

GGCCCTGGGTCCCCCTCTCGAAAGTTCCTCTGGCCGAAATTGCGCGGCGGAGACGAGGCGGGCGGAACCGTCACGGC

-112 -107 -77 -73

+1

AGAGGATTCCTTCCCCACCCTGCCTGGCCCGGCCATATATAAACAGCCACCGCCCCTCCCCGTTCCCC*ATCGCGTCT*

-54 -49 -33 -26

*CGTCTCGTGTTGTTCCCAGAACACAACCAAAATCCAAATCCTCCTCCTCCTCCCGAGCCTCGTCGATCCCTCACCCG*

*CTT****CAAG/*gta**cggcgatcctcctctcccttctcccctcgatcgattatgcgtgttccgtttccgtttccgatcgag

cgaatcgatggttaggacccatgggggacccatggggtgtcgtgtggtggtctggtttgatccgcgatatttctccg

ttcgtagtgtagatctgatcgaatccctggtgaaatcgttgatcgtgctattcgtgtgagggttcttaggtttggag

ttgtggaggtagttctgatcggtttgtaggtgagattttccccatgattttgcttggctcgtttgtcttggttagat

tagatctgcccgcattttgttcgatatttctgatgcagatatgatgaataatttcgtccttgtatcccgcgtccgta

tgtgtattaagtttgcaggtgctagttaggtttttcctactgatttgtcttatccattctgtttagcttgcaaggtt

tggtaatggtccggcatgtttgtctctatagattagagtagaataagattatctcaacaagctgttggcttatcaat

tttggatctgcatgtgtttcgcatctatatctttgcaattaagatggtagatggacatatgctcctgttgagttgat

gttgtaccttttacctgaggtctgaggaacatgcatcctcctgctactttgtgcttatacagatcatcaagattatg

cagctaatattcgatcagtttctagtatctacatggtaaacttgcatgcacttgctacttatttttgatatacttgg

atgataacatatgctgctggttgattcctacctacatgatgaacattttacaggccattagtgtctgtctgtatgtg

ttgttcctgtttgcttcagtctatttctgtttcattcctagtttattggttctctgctagatacttaccctgctggg

cttagttatcatcttatctcgaatgcattttcatgtttatagatgaatatacactcagataggtgtagatgtatgct

actgtttctctacgttgctgtaggttttacctgtggcaactgcatactcctgttgcttcgctagatatgtatgtgct

tatatagattaagatatgtgtgatggttctttagtatatctgatgatcatgtatgctcttttaacttcttgctacac

ttggtaacatgctgtgatgctgtttgttgattctgtagcactaccaatgatgaccttatctctctttgtatatgatg

tttctgtttgtttgaggcttgtgttactgctagttacttaccctgttgcctggctaatcttctg**cag/*ATG CAG***

Met Gln

***ATC TTC GTT AAG ACC CTC ACC GGC AAG ACC ATC ACC CTC GAG GTA GAG TCT TCT GAC***

Ile Phe Val Lys Thr Leu Thr Gly Lys Thr Ile Thr Leu Glu Val Glu Ser Ser Asp

B. *PvUbi2*

GAAGCCAACTAAACAAGACCATAACCATGGTGACATTTGACATAGTTGTTTACTACTTGCTTGAGCCCCACCCTTGC

-692 -625 -620

TTATCGGTTGAACATTACAAGATACACTGCGGGTGGCCTAAGGCACACCGTCCGAAACCGGCAAACCAAGCCTGATC

-568 -563

-564 -559

GCCGAAATCCAAAATCACTACCGGCAATCTCTAAAGTTTATTTCATCCTTATATGACGAGGAAAGAAAAGAAGAGAG

-514 -511

AAATAATATCTTAACTTCTAAATCAGTCGCGTCAACTTTCTCGGCTAAGAAAGTGAGCACTATCATTTCGGAGACCA

-432 -428

TGTCATGAGTGCCGACTTGCCATATCTTATTATATTCTTATTTATTTAATTATAATCCCATTGCAATACGTCTATTC

-383 -379 -321 -318

TATCATGGCCTGCCACTAACGCTCCGTCTAACGTCGTTAAGCCATTGTCATAAGCGGCTGCTCAAAACTCTTCCCGG

-296 -291 -261 -257

TGGAGGCGAGGCGTTAACGGCGTCTACAAATCTAACGGCCACCAACCATCCAGCCGCCTCTCGAAAGCTCCGCTCCG

ATCGCGGAAATTGCGTGGCGGAGACGAGCGGGCTCCTCTCACACGGCCCGGAACCGTCACGGCACGGGTGGGGGATT

-99 -95 +1

CCTTCCCCAACCCTCCCCACCTCTCCTCCCCCCGTCGCAGCCCATAAATACAGGGCCCTCCGCGCCTCTTCCCACA*A*

-48 -43 -32 -27

*TCTCACATCGTCTCATCGTTCGGAGCGCACAACCCCCGGGTTCCAAATCCAAATTGCTCTTCTCGCGACCCTCGGCG*

*ATCCTTCCCCCGCTT****CAAG/*gta**cggcgatcgtctcccccgtcctcttgccccatctcctcgctcggcgtggtttgg

tggttctgcttggtctgtggctaggaactaggctgaggcgttgacgaaatcatgctagatccgcgtgtttcctgatc

gtgggtggctgggaggtggggttttcgtgtagatctgatcggttccgctgtttatcctgtcatgctcatgtgatttg

tggggattttaggtcgtttgtccgggaatcgtggggttgcttctaggctgttcgtagatgagatcgttctcacgatc

tgctgggtcgctgcctaggttcagctaggtctgccctgtttttgggttcgttttcgggatctgtacgtgcatctatt

atctggttcgatggtgctagctaggaacaaacaactgattcgtccgatcgattgttttgttgccatgtgcaaggtta

ggtcgttatctgattgctgtagatcagagtagaataagatcatcacaagctagctcttgggcttattatgaatctgc

gtttgttgcatgattaagatgattatgctttttcttatgctgccgtttgtatatgatgcggtagcttttaactgaat

agcacacctttcctgtttagttagattagattagattgcatgatagatgaggatatatgctgctacatcagtttgat

gattctctggtacctcataatcaactagctcatgtgcttaaattgaaactgcatgtgccacatgattaagatgctaa

gattggtgaagatatatacgctgctgttcctataggatcctgtagcttttacctggtcaacatgcatcgtcctgtta

tggatagatatgcatgatagatgaagatatgtactgctacaatttgatgattcttttgtgcacctgatgatcatgca

tgctctttgcccttactttgatatacttggatgatggcatgcttagtactaatgatgtgatgaacacacatgacctg

ttggtatgaatatgatgttgctgtttgcttgtgatgagttctgtttgtttactgctaggcacttaccctgttgtctg

gttctcttttg**cag/*ATG CAG ATC TTT GTG AAG ACC CTC ACC GGC AAG ACC ATC ACC CTC***

Met Gln Ile Phe Val Lys Thr Leu Thr Gly Lys Thr Ile Thr Leu

***GAG GTG GAG TCC TCC GAC***

Glu Val Glu Ser Ser Asp

Figure S1. The sequences of the promoter candidate regions for *PvUbi1* (A) and *PvUbi2* (B). These include potential regulatory elements (underlined). The putative TATA boxes are double underlined and the transcriptional initiation site is designated as +1. Uppercase letters represent the 5’ putative promoter region containing regulatory elements. Upper case italic letters represent the 5’ untranslated sequence of the non-coding exon. Lower case letters represent the intron sequence. Upper case italic bold letters represent the exon with the corresponding amino acid displayed beneath each codon. The forward slashes represent the boundary between the exon and intron with the intron splice sites shown in bold.


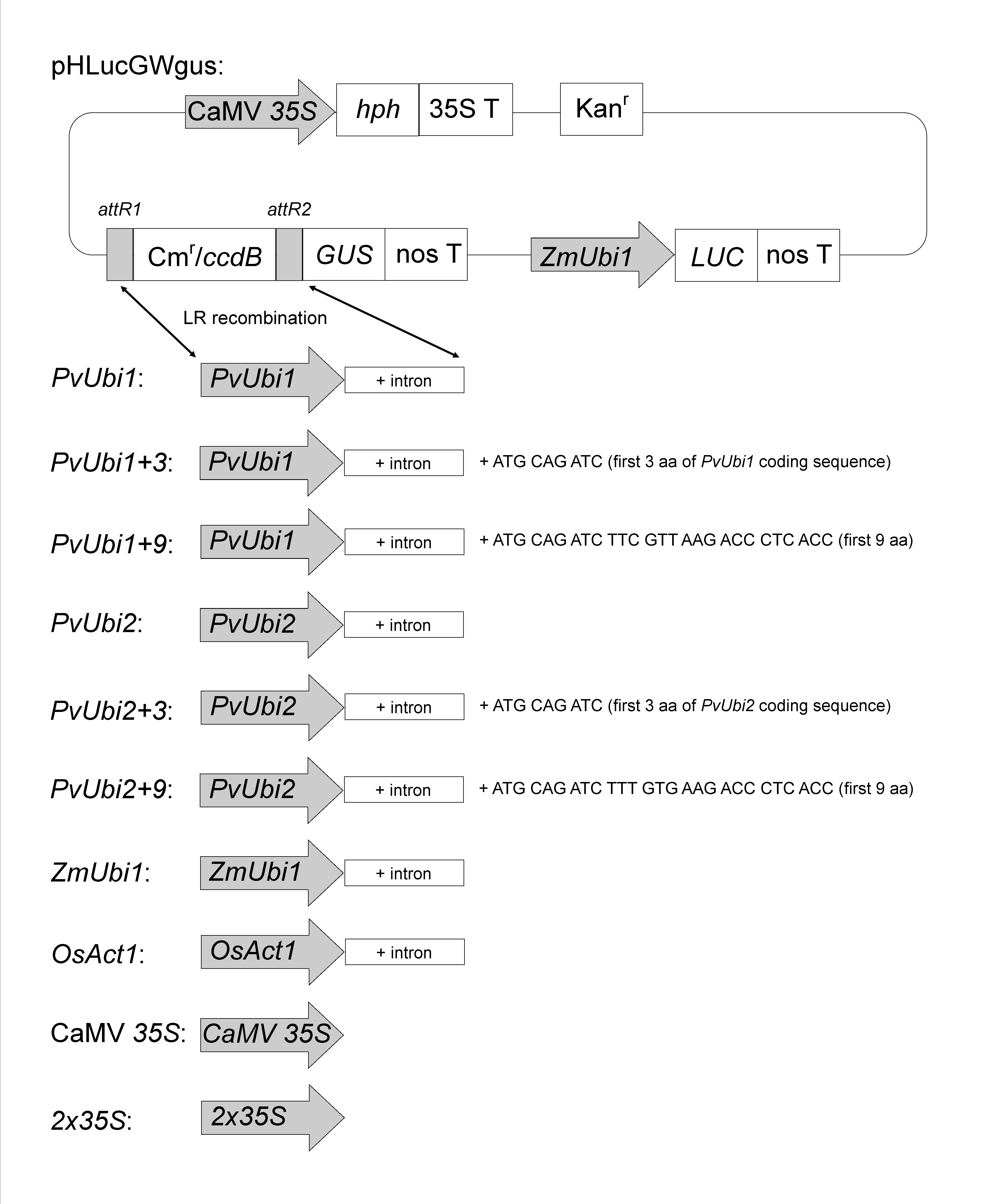


Figure S2. Schematic diagrams of the pHLucGWgus backbone used for biolistic bombardment in switchgrass and rice callus cultures. Various promoters recombined into the vector construct to drive *GUS* expression are shown in gray. Abbreviations: *PvUbi1* (switchgrass polyubiquitin 1 promoter), *PvUbi2* (switchgrass polyubiquitin 2 promoter), *ZmUbi1* (maize ubiquitin 1 promoter), *OsAct1* (rice actin 1 promoter), CaMV *35S* (cauliflower mosaic virus 35S promoter), *2x35S* (dual CaMV *35S* promoter), *GUS* (gene encoding for β-glucuronidase), *LUC* (gene encoding for luciferase), *hph* (hygromycin resistance gene), nos T (*A. tumefaciens nos* terminator sequence), 35S T (CaMV *35S* terminator), Kan^r^ (bacterial kanamycin resistance gene).


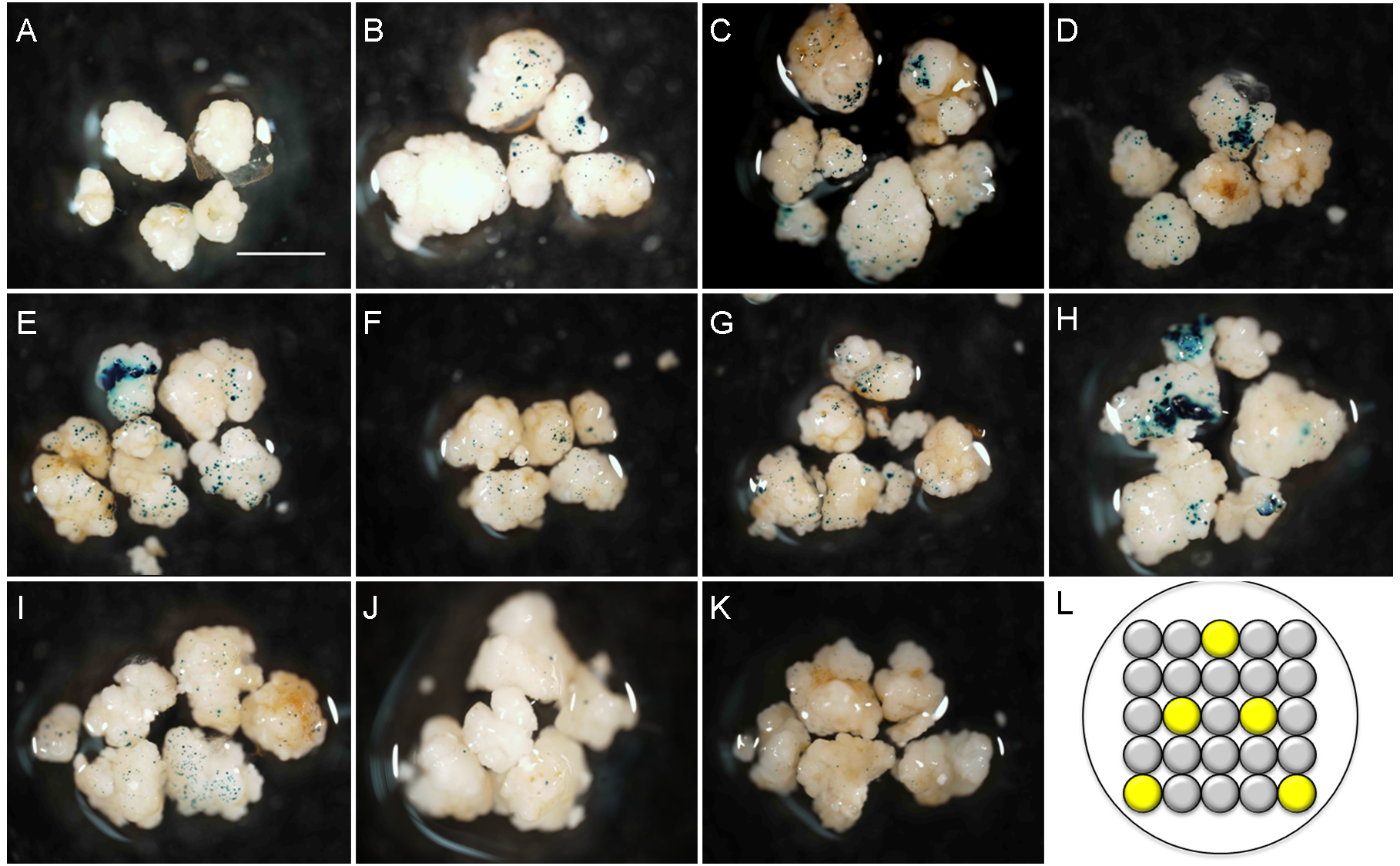


Figure S3. Representative images of rice calli after histochemical staining of GUS following biolistic transformation with no vector (A), or the pHLucGWgus construct containing the *PvUbi1* (B), *PvUbi1+3* (C)*, PvUbi1+9* (D)*, PvUbi2* (E)*, PvUbi2+3* (F)*, PvUbi2+9* (G)*, ZmUbi1* (H)*,* *OsAct1*(I), *2x35S* (J) and CaMV *35S* (K) promoters. (L) A schematic of the plate layout and the sampling distribution of calli selected for the visualization of GUS. Yellow circles represent the five calli selected per replicate plate that were bombarded for imaging. The scale bar represents 2 mm.

Table S1. Primers used in this study.

| **Amplified Region** | **Primer Name** | **Primer Sequence** | **Template** | **Experiment** |
| --- | --- | --- | --- | --- |
| *PvUbi1* gene | PvUbi1-3'R | 5’CAGCTTGGATGACCAATGGC-3' | Switchgrass cDNA | 5' RACE-PCR |
| *PvUbi1* gene | PvUbi1-3'F3 | 5’ATTTAGTGCTCCGCCTCC-3' | Switchgrass cDNA | 3' RACE-PCR |
| *PvUbi2* gene | PvUbi2-R2 | 5’GTGAGGGTCTTCACAAAGATCTGC-3' | Switchgrass cDNA | 5' RACE-PCR |
| *PvUbi2* gene | PvUbi2-5'F | 5’CTCACATCGTCTCATCGTTCGG-3' | Switchgrass cDNA | 3' RACE-PCR |
| *PvUbi1* gene | PvUbi1-RT-F4 | 5’TTGGTGCTCCGCCTGAGA-3' | Switchgrass cDNA | qRT-PCR |
|  | PvUbi1-RT-R4 | 5’ CCTGGATCTTGGCCTTCACA-3' |  |  |
| *PvUbi2* gene | PvUbi2-RT-F2 | 5’ AAGTATATGCGTCATTTGGCG-3' | Switchgrass cDNA | qRT-PCR |
|  | PvUbi2-RT-R2 | 5’ ATACTGCTGCACCACCACA-3' |  |  |
| *PvAct* gene | PvAct-RT-F1 | 5’ CAAGATTTGGAGATCCCGTG-3' | Switchgrass cDNA | qRT-PCR |
|  | PvAct-RT-R1 | 5’ AATGCTCCACGGCGAACAG-3' |  |  |
| CaMV *35S* promoter | 35S-F | 5'-AGATTAGCCTTTTCAATTTCAG-3' | pBIN-m-GFP5-er | Promoter Validation |
|  | 35S-R | 5'-CGTGTTCTCTCCAAATGAAA-3' |  |  |
| *2x35S* promoter | d35S-F | 5'-GGTCAACATGGTGGAGCACGAC-3' | pMDC32 | Promoter Validation |
|  | d35S-R | 5'-GGGATCCTCTAGAGTCGAGGTCC-3' |  |  |
| *OsAct1* promoter | OsAct1-F | 5'-CTCGAGGTCATTCATATGCT-3' | pCOR113 | Promoter Validation |
|  | OsAct1-R | 5'-TCTACCTACAAAAAAGCTCC-3' |  |  |
| *ZmUbi1* promoter | ZmUbi1-F | 5'-TGCAGTGCAGCGTGACCCGG-3' | pAHC25 | Promoter Validation |
|  | ZmUbi1-R | 5'-TGCAGAAGTAACACCAAACAACAGGG-3' |  |  |
| *PvUbi1* promoter | PvUbi1-F | 5'-CCACTGGAGAGGGGCACACACG-3' | Pv9G7 contig | Promoter Validation |
|  | PvUbi1-R | 5'-CTGCAGAAGATTAGCCAGGCAACAGG-3' |  |  |
| *PvUbi1+3* promoter | PvUbi1-F | 5'-CCACTGGAGAGGGGCACACACG-3' | Pv9G7 contig | Promoter Validation |
|  | PvUbi1+3-R | 5'-GATCTGCATCTGCAGAAGATTAGCCAGG-3' |  |  |
| *PvUbi1+9* promoter | PvUbi1+9-F | 5'-CCACTGGAGAGGGGCACACACGTCAGTG-3' | Pv9G7 contig | Promoter Validation |
|  | PvUbi1+9-R | 5'-GGTGAGGGTCTTAACGAAGATCTGCATCTGCAG-3' |  |  |
| *PvUbi2* promoter | PvUbi2-F | 5'-GAAGCCAACTAAACAAGACCATAACCATGGTG-3' | Pv9G7 contig | Promoter Validation |
|  | PvUbi2-R | 5'-CTGCAAAAGAGAACCAGACAACAGGG-3' |  |  |
| *PvUbi2+3* promoter | PvUbi2-F | 5'-GAAGCCAACTAAACAAGACCATAACCATGGTG-3' | Pv9G7 contig | Promoter Validation |
|  | PvUbi2+3-R | 5'-GATCTGCATCTGCAAAAGAGAACCAGAC-3' |  |  |
| *PvUbi2+9* promoter | PvUbi2-F | 5'-GAAGCCAACTAAACAAGACCATAACCATGGTG-3' | Pv9G7 contig | Promoter Validation |
|  | PvUbi2+9-R | 5'-GGTGAGGGTCTTCACAAAGATCTGCATC-3' |  |  |
